# Analysis of SARS-CoV-2 genome evolutionary patterns

**DOI:** 10.1128/spectrum.02654-23

**Published:** 2024-01-10

**Authors:** Shubhangi Gupta, Deepanshu Gupta, Sonika Bhatnagar

**Affiliations:** 1 Department of Biological Sciences and Engineering, Computational and Structural Biology Laboratory, Netaji Subhas University of Technology, Dwarka, New Delhi, India; 2 Division of Biotechnology, Computational and Structural Biology Laboratory, Netaji Subhas Institute of Technology, Dwarka, New Delhi, India; University of Nevada Reno, Reno, Nevada, USA

**Keywords:** SARS-CoV-2, genome, mutation rates, selection pressure, evolution, vaccination

## Abstract

The spread of SARS-CoV-2 virus accompanied by public availability of abundant sequence data provides a window for the determination of viral evolutionary patterns. In this study, SARS-CoV-2 genome sequences were collected from seven countries in the period January 2020–December 2022. The sequences were classified into three phases, namely, pre-vaccination, post-vaccination, and recent period. Comparison was performed between these phases based on parameters like mutation rates, selection pressure (d*N*/d*S* ratio), and transition to transversion ratios (T*i*/T*v*). Similar comparisons were performed among SARS-CoV-2 variants. Statistical significance was tested using Graphpad unpaired *t*-test. The analysis showed an increase in the percent genomic mutation rates post-vaccination and in recent periods across all countries from the pre-vaccination sequences. Mutation rates were highest in NSP3, S, N, and NSP12b before and increased further after vaccination. NSP4 showed the largest change in mutation rates after vaccination. The d*N*/d*S* ratios showed purifying selection that shifted toward neutral selection after vaccination. N, ORF8, ORF3a, and ORF10 were under highest positive selection before vaccination. Shift toward neutral selection was driven by E, NSP3, and ORF7a in the after vaccination set. In recent sequences, the largest d*N*/d*S* change was observed in E, NSP1, and NSP13. The T*i*/T*v* ratios decreased with time. C→U and G→U were the most frequent transitions and transversions. However, U→G was the most frequent transversion in recent period. The Omicron variant had the highest genomic mutation rates, while Delta showed the highest d*N*/d*S* ratio. Protein-wise d*N*/d*S* ratio was also seen to vary across the different variants.

**IMPORTANCE:**

To the best of our knowledge, there exists no other large-scale study of the genomic and protein-wise mutation patterns during the time course of evolution in different countries. Analyzing the SARS-CoV-2 evolutionary patterns in view of the varying spatial, temporal, and biological signals is important for diagnostics, therapeutics, and pharmacovigilance of SARS-CoV-2.

## INTRODUCTION

Coronavirus disease 2019 (COVID-19) emerged as an epidemic with its initial spread in Wuhan (China) and was declared as a pandemic in March 2020 by the World Health Organization. Over 650 million confirmed cases of COVID-19, with more than 6 million deaths have been reported worldwide by WHO as of 15 May 2022 (1). In India, the first case of COVID-19 was reported in January 2020. An exponential increase in the number of cases from March 2020 to September 2020 marked the first wave. The second wave was marked by a sudden increase in COVID-19 cases from 12 March 2021 and high death rates (2). The Omicron variant of the causative SARS-CoV-2 characterized the third wave of COVID-19 with higher transmissibility but lower death rates (3).

SARS-CoV-2 has continuously evolved over time, resulting in numerous genetic variants across the globe (4). These variants are classified into three main categories, i.e., variant under monitoring (VUM), variant of interest (VOI), and variant of concern (VOC) (5). The VUM includes the GH/G (S gene D614G variant) progeny. VOI includes the Mu (lineage B.1.621) and Lambda (C.37), whereas VOC includes the alpha (lineage B.1.1.7), beta (lineage B.1.351), gamma (lineage P.1), delta (lineage B.1.617.2), and omicron variants (lineages BA1, BA2, BA4, and BA5) (5, 6).

The S protein is responsible for SARS-CoV-2 attachment and entry by binding to the ACE2 (7–10). Continuous mutations in the Spike increase virus adaptability for escaping vaccine treatment resulting in high survival rates and spread of the virus (11–14). Multiple mutations in the omicron variant S protein may also influence its interaction with ACE2, thereby leading to antibody escape (15, 16). Mutations in the S region of SARS-CoV-2 have led to epitope loss, resulting in escape from the vaccine treatment, the most frequent mutation being D614G (17, 18). D614G is also found in the S region of clades G/GR/GRY/GH/GV that has high human host infectivity rate due to efficient transmission (19, 20).

The SARS-CoV-2 genome contains 14 ORFs. Of these, *ORF1a* encodes the NSPs 1–11, while *ORF1b* encodes NSP12-16. Together, NSPs 1–16 form the replicase-transcriptase complex. This is followed by 13 ORFs encoding the four main structural proteins, namely, S, E, M, N, and interspersed by 9 accessory factors (21). Various studies have been performed on genomic mutations in the SARS-CoV-2 virus. The ratio of d*N*/d*S* > 1 indicates positive selection and has been reported in the *S* glycoprotein (22, 23). Comparative analysis of SARS-CoV-2, SARS-CoV, and MERS-CoV showed a positive evolution model along with higher d*N*-d*S* due to the dominance of d*N* (24). Studies of mutations in the diagnostic targets in COVID-19 have suggested that the *N* gene has the highest number of mutations (25, 26).

Analysis of 469 genome sequences from Indian patients led to the identification of 536 d*N* and d*S* mutations in the six genes; *ORF1ab*, *S*, *N*, *ORF3a*, *ORF7a*, and *ORF8* (27). A broad analysis showed 33 different mutations in 837 Indian SARS-CoV-2 whole-genome sequence isolates, of which 18 were unique to India. S, N, NSP3, NSP12, and NSP2 coding genes showed novel mutations and d*N* was found to be more than d*S* by approximately threefold (28). Modeling of the epidemic with different strains and mutations showed the emergence of a virus with higher transmissibility and evolutionary adaptations (29).

In this study, we calculated the genomic rates of mutation and d*N*/d*S* ratios in SARS-CoV-2 genome sequences taken from seven countries and showed that they increased with time, whereas the T*i*/T*v* decreased. Similarly, these parameters were also estimated for different known SARS-CoV-2 variants, where Omicron variant sequences showed highest mutation rate as compared to the other variants, but delta showed the highest d*N*/d*S* ratio. The highest mutating protein along with their individual d*N*/d*S* ratios and mutation rates were determined within each country. NSP3, S, N, and NSP12b had the highest genomic mutation rates both in before and after vaccination phase. NSP3 showed highest genomic mutation rates before vaccination, which was replaced by S in the after vaccination, while a significant rise was observed in the NSP4 genomic mutation rate. N, ORF8, ORF3a, and ORF10 were under strong positive selection pressure before vaccination, whereas after vaccination, E, ORF7a, and NSP3 showed the highest increase in the d*N*/d*S* ratio. The recent sequences showed the highest d*N*/d*S* ratios in E, NSP1, and NSP13. The estimated properties showed similar patterns in genetic variability across all geographic regions despite the use of different vaccine technologies. This implies that the forces of evolution have been uniform across multiple parameters. However, there is a definite change in the pattern of mutations with time. While the highly mutating and positively selected genes are important for pharmacovigilance and vaccination, the negatively selected ones are important for diagnostics. This is the only study that has compared these different mutational parameters comprehensively with time and geographical regions, and in different variants both at the whole genome and gene level.

## MATERIALS AND METHODS

### Collection of genomic sequences of SARS-CoV-2

To perform a critical analysis of the data, genome sequences of SARS-CoV-2 were retrieved in FASTA format for different countries using the GISAID database (30, 31). The countries taken into consideration included India, England, Canada, Italy, France, USA (Washington), and the Netherlands. Additionally, genome sequences of different SARS-CoV-2 variants like Alpha, Beta, Gamma, Lambda, Mu, Delta, and Omicron were also retrieved.

The accuracy of genome sequences retrieved from the database was ensured using filters like complete sequence, high coverage, and complete collection date. The sequences downloaded were high coverage, having <1% Ns (unidentified nucleotides) and <0.05% unique amino acid mutations. In all the sequences, insertions and deletions were accepted only when verified by the submitter.

### Classifying the genome sequences retrieved

SARS-CoV-2 genome sequences submitted in the GISAID database from the seven countries were downloaded from three distinct periods. The genome sequences were classified into three categories as follows:

- The sequences considered pre-vaccination sequences had the sample collection date of at least 1 month before the start date of vaccination taken from the website of WHO and from the Government vaccination websites of the concerned countries. The data sets were taken in triplicates.
- The start date of vaccination was different for each country considered. After the date of start of vaccination, a buffer period is required for the vaccine to reach the population. Therefore, sequences collected 5–6 months after the date of start of vaccination were considered as after vaccination sequences. The data sets were taken in triplicates.
- A set of sequences that constitute a recent data set were also taken. As the available number of recent sequences were less in number for many countries, triplicate data sets for this period could not be taken.
- Additionally, approximately 1,000 sequences of each SARS-CoV-2 variant were obtained using the “Variant” filter of GISAID.

### Sequence alignment and mutational data analysis

The first reported sequence, Wuhan-Hu-1 (Accession ID: NC045512), was taken as the reference for pre-vaccination, post-vaccination, and recent sequences. Thus, Wuhan-Hu-1 sequence was added as a reference for the alignment. The mutation rates of all the genomic sequences were calculated using COVID-19 genome annotator, an online web-based tool that aligns the input sequences in “nucmer” alignment tool and processes the alignment output using UNIX along with R scripts (32). The aligned sequences were compared with the reference genome (Wuhan-Hu-1) and amongst each other to find information about the mutational sites. The Jupyter notebook (33) was used in association with the “pandas” python library (34) to visualize and analyse the data obtained. The following values were calculated:

- Percentage mutation rate:
  The mutation rate has been calculated using the following equation:

$Mutation\;Rate\;\left(\%\right)=\;\frac{Total\;no.\;of\;nucleotide\;mutations}{lg*gs}\;\;\times 100$

  where “lg” refers to the length of the data set taken, “gs” refers to the length of the genome sequence and the total no. of mutations estimated with respect to number of single nucleotide mutations (35). Mutation rate is the measure that refers to the frequency of mutations per generation in the population or in an organism (36).
- Selection pressure has been calculated as the d*N*/d*S* ratio. If d*N*/d*S* ratio exceeds unity (d*N*/d*S* > 1), the mutations are said to be occurring under positive selection which promotes the accumulation of beneficial mutations, whereas if d*N*/d*S* ratio is below unity (d*N*/d*S* < 1), the mutations are said to be occurring under negative selection that promotes mutations that are favouring selective removal of deleterious alleles (37, 38).
  A mutation table was obtained after the multiple sequence alignment was performed by the COVID-19 genome annotator. The table included the varclass information, i.e., the type/class of mutation that occurred in the input sample sequences. Under varclass, the mutations described as SNP were taken as observed non-synonymous mutations (*N*_m_), whereas SNP_silent were taken observed synonymous mutations (*S*_m_).
  Further, the selection pressure d*N*/d*S* were determined by normalizing the estimated *N*_m_ and *S*_m_ with the overall expected number of non-synonymous (*N*_ref_) and synonymous (*S*_ref_) sites in the Wuhan-Hu-1 reference genome. *N*_ref_ and *S*_ref_ sites were determined using a biopython script in which the codon substitution table from *T. gojobori* was implemented (39). Therefore, d*N*/d*S* ratio was calculated as:

$\frac{dN}{dS}\;=\;\frac{N_{m}/N_{ref}}{S_{m}/S_{ref}}$

- The mutation table obtained from the COVID-19 genome annotator also provided information about mutation type/class (varclass) occurring in the respective protein region (protein). Both the “varclass” and “protein” information was considered together to calculate the number of SNP (*N*_m_) and SNP_silent (*S*_m_) for each SARS-CoV-2 protein.
  Furthermore, the selection pressure d*N*/d*S* of each protein were determined by normalizing the estimated *N*_m_ and *S*_m_ of the protein with their respective expected number of non-synonymous (*N*_ref_) and synonymous (*S*_ref_) sites within the Wuhan-Hu-1 reference genome. These *N*_ref_ and *S*_ref_ sites were determined using a biopython script. Therefore, d*N*/d*S* ratio was calculated as

$\left(\frac{dN}{dS}\right)of\;protein\;=\;\frac{\left(\frac{N_{m}}{N_{ref}}\right)of\;the\;protein}{\left(\frac{S_{m}}{S_{ref}}\right)of\;the\;protein}$

- The T*i*/T*v* in the RNA virus has been calculated as the ratio of T*i* (C↔U and A↔G) to T*v* (A↔C, A↔U, G↔C, and G↔U), determined for the pre-, post-, and recent vaccination sequence groups using the data obtained from the COVID-19 genome annotator. The mutation table data from the genome annotator provided the refvar (sequence at the mutation site on reference genome) and qvar (sequence at the mutation site on sample sequence) mutation information. The number of T*i* mutations C-U, U-C, A-G, and G-A was counted using Microsoft Excel. Similarly, C-A, A-C, U-A, A-U, G-U, U-G, C-G, and G-C changes were counted as the total number of T*v*. Therefore, the T*i*/T*v* ratio was calculated as:

$Ti/Tv\;=\;\frac{Total\;no.\;of\;transitions}{Total\;no.\;of\;transversions}$

### Statistical analysis

The statistical significance of the differences between the means was carried out. The unpaired *t*-test was performed on the % mutation rates, selection pressure and T*i*/T*v* ratios using GraphPad QuickCalcs (https://www.graphpad.com/quickcalcs/ttest1.cfm).

## RESULTS AND DISCUSSION

In this work, we have carried out comparison of the SARS-CoV-2 genome sequences from seven different demographic regions, namely, India, France, England, Canada, Italy, Netherlands, and USA (Washington, D.C.). A total of 74,870 retrieved sequences were classified into three different time periods:

- Pre-vaccination phase: Broadly, the pre-vaccination sequences taken were ranging from January 2020 to December 2020 (GISAID Identifier: EPI_SET_230517 nt).
- Post-vaccination phase: The post-vaccination sequences taken were ranging from May 2021 to April 2022 (GISAID Identifier: EPI_SET_230517va).
- Recent period: The recent period sequences were taken from June 2022 to December 2022 (GISAID Identifier: EPI_SET_230518oq).
- Additionally, a total of 7,209 sequences of different variants were also analyzed. As new variants emerged sequentially over time, collection dates of the sequences considered for the SARS-CoV-2 variants ranged from November 2020 to January 2022 (GISAID Identifier: EPI_SET_230518xv).

The sequences were compared for % mutation rates, d*N*/d*S* ratio and T*i*/T*v* ratio. The analysis was carried out for the whole genome as well as for every individual gene.

### Evolutionary patterns in different geographical locations and time periods

**Fig 1:**
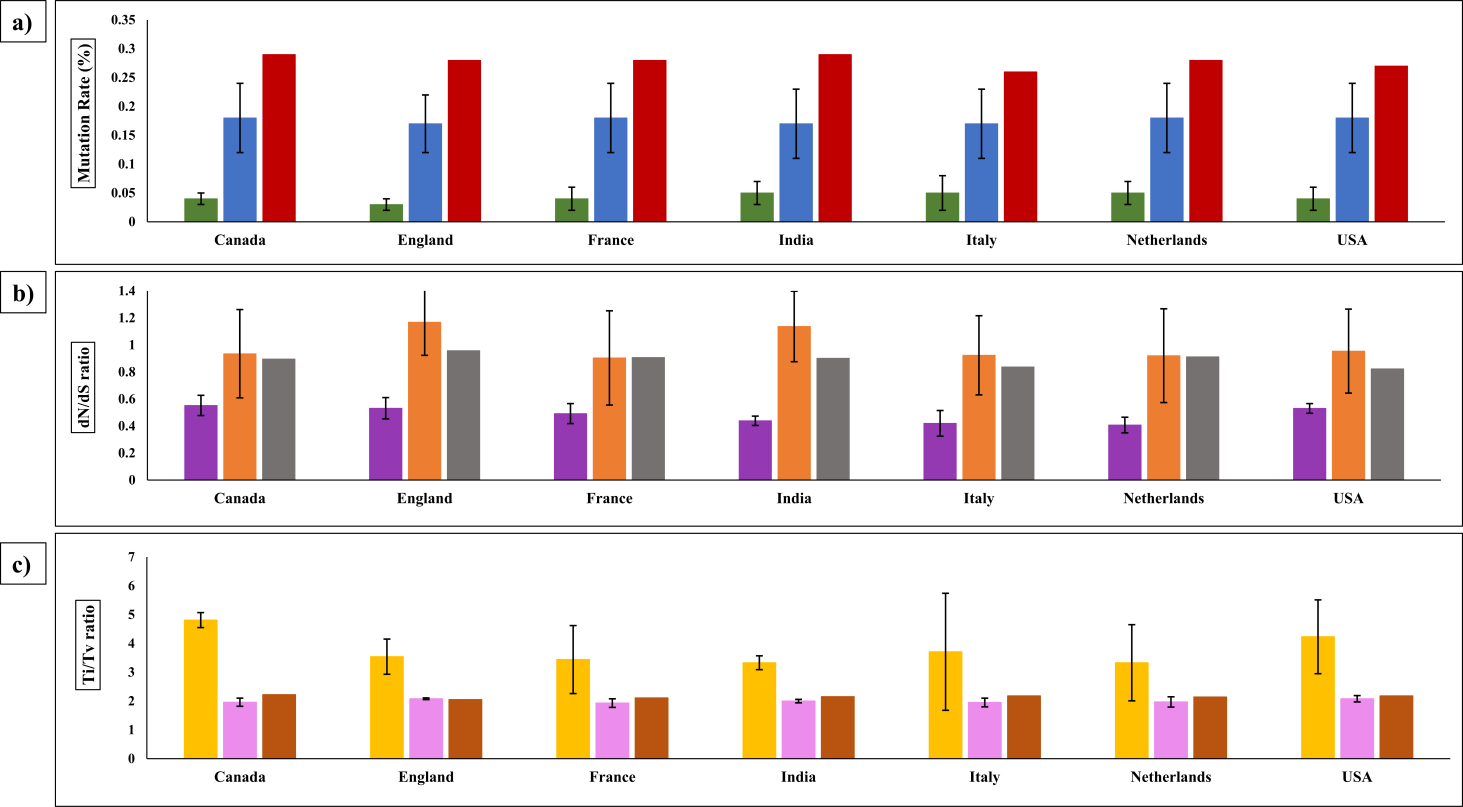
Comparison of mutation rates, d*N*/d*S* and T*i*/T*v* within the countries in the three phases. (**a**) Mutation rates with respect to before (green), after vaccination (blue), and recent period (red); (**b**) Selection pressure (d*N*/d*S* ratio) with respect to before (violet), after vaccination (orange), and recent period (gray); (**c**) Transition to transversion (T*i*/T*v*) ratio with respect to before (yellow), after vaccination (pink), and recent period (brown). Black margins in the graph represent the standard deviation in the before and after vaccination.

- Increase in mutation rates: The % genomic mutation rates estimated for each country in the pre-vaccination, post-vaccination, and the recent period are listed in Table 1. The genome mutation rates were calculated as (0.04 ± 0.02) % in the pre-vaccination phase. The average mutation rates increased to (0.17 ± 0.05) % in the post-vaccination period. A comparable increase was seen in all the seven countries studied. The mutation rates for sequences from a more recent interval were higher than the post-vaccination period. The mutation rate was found to be (0.28 ± 0.01) %, consistently across all the seven countries. Thus, there was an average increase of three- to fourfold in the % genomic mutation rates in all the countries after vaccination and six- to sevenfold increase in the recent period in comparison with mutation rates in the pre-vaccination sequences. Figure. 1a shows the comparison of percent mutation rates within the countries in pre-vaccination, post-vaccination, and recent period. There was a similar increase in the % genomic mutation rates in the post-vaccination period in comparison with the pre-vaccination period across all the seven countries studied. This has increased further in the recent sequences. Despite the large variation in mutation rates in each country, there was a definite increase in the post-vaccination sequences especially in the post-vaccination period. In the recent sequences, the mutation rates show a still higher trend in all the seven countries studied. The significance of the difference between the means was verified using unpaired *t*-test (*P*-value 0.0001; confidence interval of 95%) and is shown in Table 2. The differences in percent genomic mutation rates were found to be extremely statistically significant between the three phases with a two-tailed *P*-value of less than 0.0001.

**TABLE 1:** Data comparison of different countries before and after vaccination along with the recent data

Countries	India	France	England	Canada	Italy	Netherlands	USA (Washington DC)	Total sequences
Properties								Average of properties
**Date of start of vaccination**	16/01/2021	27/12/2020	08/12/2020	14/12/2020	27/12/2020	06/01/2021	17/12/2020	**–**
**Vaccine used (Technology**)	Covishield/Covaxin	Pfizer	Pfizer	Pfizer	Pfizer	Pfizer	Pfizer/Moderna	**–**
No. of sequences before vaccination (Jan 2020)–(1Dec 2020)	5,022	5,071	5,114	5,178	4,531	4,964	5,090	34,970
No. of sequences after vaccination (May 2021–April 2022)	5,270	5,282	5,070	5,199	4,953	5,029	5,217	36,020
No. of recent sequences (June 2022–Dec 2022)	572	531	555	591	540	533	558	3,880
% Mutation rates (before)	0.05 ± 0.02	0.04 ± 0.02	0.03 ± 0.01	0.04 ± 0.01	0.05 ± 0.03	0.05 ± 0.02	0.04 ± 0.02	0.04 ± 0.02
% Mutation rates (after)	0.17 ± 0.06	0.18 ± 0.06	0.17 ± 0.06	0.18 ± 0.06	0.17 ± 0.06	0.18 ± 0.06	0.18 ± 0.06	0.17 ± 0.05
% Mutation rates (recent)	0.29	0.28	0.28	0.29	0.26	0.28	0.27	0.28 ± 0.01
**Ratio of mutation rates (after/before**)	**3.40**	**4.50**	**5.67**	**4.50**	**3.40**	**3.60**	**4.50**	**4.22 ± 0.76**
**Ratio of mutation rates (recent/before**)	**5.80**	**7.00**	**9.33**	**7.25**	**5.20**	**5.60**	**6.75**	**6.70 ± 1.28**
d*N*/d*S* ratio (before)	0.44 ± 0.03	0.50 ± 0.07	0.53 ± 0.08	0.55 ± 0.07	0.42 ± 0.09	0.40 ± 0.06	0.53 ± 0.04	0.48 ± 0.06
d*N*/d*S* ratio (after)	1.13 ± 0.26	0.90 ± 0.35	1.17 ± 0.24	0.94 ± 0.33	0.92 ± 0.30	0.92 ± 0.35	0.95 ± 0.31	0.99 ± 0.11
d*N*/d*S* ratio (recent)	0.90	0.91	0.96	0.90	0.84	0.91	0.82	0.90 ± 0.05
**d*N*/d*S* ratio (after/before**)	**2.57**	**1.80**	**2.20**	**1.71**	**2.20**	**2.30**	**1.80**	**2.08 ± 0.32**
**d*N*/d*S* ratio (recent/before**)	**2.04**	**1.82**	**1.81**	**1.64**	**2.00**	**2.27**	**1.55**	**1.88 ± 0.25**
T*i*/T*v* ratio (before)	3.33 ± 0.24	3.44 ± 1.18	3.54 ± 0.61	4.81 ± 0.26	3.71 ± 2.03	3.33 ± 1.32	4.23 ± 1.28	3.77 ± 1.10
T*i*/T*v* ratio (after)	2.00 ± 0.06	1.93 ± 0.15	2.08 ± 0.03	1.96 ± 0.14	1.95 ± 0.15	1.97 ± 0.18	2.08 ± 0.11	2.00 ± 0.12
T*i*/T*v* ratio (recent)	2.15	2.11	2.05	2.22	2.18	2.14	2.18	2.15 ± 0.05
**T*i*/T*v* ratio (after/before**)	**0.60**	**0.56**	**0.59**	**0.41**	**0.53**	**0.60**	**0.50**	**0.54 ± 0.06**
**T*i*/T*v* ratio (recent/before**)	**0.64**	**0.61**	**0.58**	**0.46**	**0.58**	**0.64**	**0.51**	**0.57 ± 0.06**
% GC content (before)	37.93 ± 0.03	37.96 ± 0.02	37.92 ± 0.05	37.97 ± 0.01	37.92 ± 0.02	37.96 ± 0.02	37.95 ± 0.04	37.94 ± 0.02
% GC content (after)	37.81 ± 0.06	37.85 ± 0.09	37.84 ± 0.07	37.92 ± 0.03	37.84 ± 0.08	37.88 ± 0.08	37.81 ± 0.10	37.85 ± 0.07
% GC content (recent)	37.81	37.82	37.76	37.89	37.76	37.82	37.85	37.82 ± 0.05

^*a*^ The prominent results reflecting change in mutation rates (%), d*N*/d*S* and T*i*/T*v* ratio from the pre-vaccination period for each country are shown in bold along with the overall change.

**TABLE 2:** Unpaired *t*-test calculations for the significance testing of the determined country-wise parameters

Group Comparison	Parameters of comparison	*t*-value	Degrees of freedom (df)	Mean difference	Standard error of difference	95% confidence interval (C.I.)	Two-tailed *P*-value
Pre-vaccination to Post vaccination period	Mutation rates	452.3	70,988	−0.13	0.000	−0.1306 to −0.1294	<0.0001
	d*N*/d*S* ratio	763.7		−0.51	0.001	−0.5113 to −0.5087	<0.0001
	T*i*/T*v* ratio	303.5		1.77	0.006	1.7585 to 1.7815	<0.0001
	% GC content	231.4		0.09	0.000	0.0892 to 0.0908	<0.0001
Post-vaccination and Recent period	Mutation rates	136.7	39,898	−0.11	0.001	−0.1116 to −0.1084	<0.0001
	d*N*/d*S* ratio	50.4		0.09	0.002	0.0865 to 0.0935	<0.0001
	T*i*/T*v* ratio	77.1		−0.15	0.002	−0.1538 to −0.1462	<0.0001
	% GC content	25.9		0.03	0.001	0.0277 to 0.0323	<0.0001
Pre-vaccination and Recent period	Mutation rates	737.3	38,848	−0.24	0.000	−0.2406 to −0.2394	<0.0001
	d*N*/d*S* ratio	420.1		−0.42	0.001	−0.4220 to −0.4180	<0.0001
	T*i*/T*v* ratio	91.7		1.62	0.018	1.5853 to 1.6547	<0.0001
	% GC content	287.2		0.12	0.000	0.1192 to 0.1208	<0.0001

In RNA viruses, proofreading incapability of RNA polymerases accounts for their high mutation rates. Natural selection further results in increased adaptability and faster replication of viruses referring to their high mutation rates (40, 41). However, the highly conserved nsp14 in the *Coronaviridae family* has a proofreading function that may be a crucial factor for variation in the large and complex viral genome (42). Absolute estimates of mutation rates in coronaviruses ranged from 0.67 to 1.33 × 10^−5^ per site per year in infectious bronchitis virus to 0.44–2.77 × 10^−2^ per site per year in mouse hepatitis virus. Comparatively, the global SARS-CoV-2 mutation rate was estimated to be moderate at 6 × 10^−4^ per site per year (43). The more than twofold increase in mutation rates with time in SARS-CoV-2 as observed in this study would bring the genomic mutation rates at par with the reported genomic mutation rates for non-coronaviruses and the other RNA viruses like Influenza virus and HIV-1 (44).
  - Mutation rates in SARS-CoV-2 proteins: The pattern of mutations in proteins within the SARS-CoV-2 genome sequences was analyzed for each protein in the pre-vaccination, post-vaccination, and recent period (Table 3). As shown, NSP3, S, N, and NSP12b coding regions had the highest % mutation rates in the pre-vaccination period. There was little variation observed in the highest mutating genes in different countries studied. Previously, NSP3 was reported as the mutation hotspot of SARS-CoV-2 genome and mostly found to be co-mutating with NSP12b due to its involvement in replication and transcription complexes (45–49). However, in the post-vaccination and recent period, the highest mutating genes remained the same, where *S* was observed to uniformly have the highest % mutation rates followed by *N* and NSP3. From the pre-vaccination sequences, *S* showed gradual increase in the % mutation rates till the recent period across all the countries. *S* contains the receptor-binding domain (RBD) which is highly variable, and through its mutational changes, it is known to affect viral replication, transmission and is also involved in immune escape (50, 51). In comparison with *S*, *N* has lower mutation rates and is comparatively stable (52). *N* is considered as a vital hotspot for mutations mainly in its serine-rich domain due to its involvement in viral replication and packaging (53, 54) and can be considered a novel target for vaccine design (55). Comparison of mutation patterns of all the proteins in Table 4 showed that NSP4 had shown low mutation rates before vaccination but rose to be the fifth highest mutating protein post-vaccination. In recent sequences, it uniformly occupied the fourth position and overtook NSP12b in all the countries. NSP4 along with NSP6 and NSP3 transmembrane proteins, induce the development of double membrane vesicles (DMVs) by reorganizing the endoplasmic reticulum of the host cell, and therefore it may be co-mutating with NSP3 (56, 57). In contrast with this, ORF10, NSP7, and NSP10 were the lowest mutating in all the three periods for all the countries taken into consideration. This was in line with previous studies where they were found to be conserved within the SARS-CoV-2 genome with few or no mutations (47, 58–60).

- Increasing Selection pressure: The d*N*/d*S* ratio in each country for the pre-vaccination, post-vaccination and the recent period are listed in Table 1. The global d*N*/d*S* ratio in the SARS-CoV-2 genome during the pre-vaccination period was calculated as 0.48 ± 0.06, thus indicating a purifying selection pressure. The observed value is similar to previous studies showing that SARS-CoV-2 genome depicts overall purifying selection pressure (61–63) amd study reporting overall SARS-CoV-2 genome dN/dS of around 0.55 (61). The d*N*/d*S* ratio increased to 0.99 ± 0.11 in the post-vaccination period in various demographic regions, suggesting SARS-CoV-2 genomes showing overall neutral selection. The recent period sequences showed an average d*N*/d*S* of 0.90 ± 0.05. The comparison of d*N*/d*S* ratios in all the countries for pre-vaccination, post-vaccination and recent period is shown in Fig. 1b. There was more variation in the d*N*/d*S* value of sequences taken from the post-vaccination period in comparison before vaccination. Taken together, a definite increase in d*N*/d*S* is seen in post-vaccination sequences but the data from recent sequences was inconclusive because of small sample size. The differences in the mean d*N*/d*S* ratios were found to be extremely statistically significant between the three phases with a unpaired two-tailed *t*-test *P*-value of less than 0.0001 (Table 2).

**TABLE 3:** Data comparison of highly mutating genes before, after vaccinations, and recent period in each country along with their respective selection pressures

Country	Before vaccination (Jan 2020)–(Dec 2020)			After vaccination (May 2021–April 2022)			Recent period (June 2022– Dec 2022)		
	Highly mutating proteins	Selection pressure (d*N*/d*S*)	Mutation rate (%)	Highly mutating proteins	Selection pressure (d*N*/d*S*)	Mutation rate (%)	Highly mutating proteins	Selection pressure (d*N*/d*S*)	Mutation rate (%)
India	NSP3 S NSP12b	0.16 ± 0.02 0.63 ± 0.02 1.35 ± 0.31	0.009 ± 0.002 0.007 ± 0.003 0.005 ± 0.001	fruiS NSP3 N	5.00 ± 1.00 0.44 ± 0.14 5.30 ± 1.93	0.05 ± 0.04 0.02 ± 0.002 0.01 ± 0.002	S NSP3 NSP4	8.56 0.26 1.04	0.12 0.03 0.02
England	NSP3 S NSP12b	0.1 ± 0.03 1.27 ± 0.37 1.28 ± 0.80	0.005 ± 0.003 0.005 ± 0.002 0.004 ± 0.001	S NSP3 N	6.08 ± 0.80 0.56 ± 0.26 9.80 ± 4.6	0.05 ± 0.04 0.02 ± 0.002 0.01 ± 0.001	S NSP3 N	7.12 0.22 2.50	0.11 0.03 0.02
Canada	NSP3 S NSP12b	0.08 ± 0.09 1.99 ± 1.10 1.57 ± 0.74	0.007 ± 0.004 0.005 ± 0.001 0.004 ± 0.001	S NSP3 N	6.16 ± 2.06 0.35 ± 0.14 3.52 ± 2.05	0.06 ± 0.04 0.02 ± 0.003 0.01 ± 0.002	S NSP3 NSP4	6.58 0.28 1.01	0.12 0.03 0.02
France	NSP3 NSP12b S	0.08 ± 0.04 1.71 ± 1.18 3.60 ± 0.64	0.007 ± 0.003 0.006 ± 0.002 0.005 ± 0.002	S NSP3 N	4.4 ± 0.73 0.43 ± 0.16 6.13 ± 2.04	0.05 ± 0.04 0.02 ± 0.003 0.01 ± 0.002	S NSP3 N	6.02 0.26 1.95	0.11 0.02 0.02
Italy	S NSP3 NSP12b	2.17 ± 0.25 0.09 ± 0.04 1.71 ± 1.18	0.009 ± 0.005 0.009 ± 0.004 0.005 ± 0.001	S NSP3 N	4.27 ± 0.50 0.41 ± 0.15 5.96 ± 2.00	0.05 ± 0.04 0.02 ± 0.003 0.01 ± 0.002	S NSP3 N	5.46 0.26 2.11	0.10 0.02 0.02
Netherlands	NSP3 S NSP12b	0.09 ± 0.02 2.24 ± 0.74 1.07 ± 0.45	0.009 ± 0.004 0.007 ± 0.003 0.007 ± 0.003	S NSP3 N	4.50 ± 0.42 0.37 ± 0.11 4.73 ± 2.27	0.06 ± 0.04 0.02 ± 0.003 0.01 ± 0.001	S NSP3 N	6.36 0.30 2.61	0.11 0.02 0.02
USA (Washington DC)	NSP3 S NSP12b	0.09 ± 0.03 2.01 ± 1.21 1.05 ± 0.46	0.005 ± 0.003 0.004 ± 0.003 0.004 ± 0.003	S NSP3 N	5.21 ± 1.14 0.40 ± 0.14 4.14 ± 3.35	0.05 ± 0.04 0.02 ± 0.003 0.01 ± 0.002	S NSP3 NSP4	4.77 0.28 1.04	0.11 0.02 0.02

**TABLE 4:** Highest (top) to lowest (bottom) protein-wise contribution to mutation rates in all the countries before, after vaccination and in the recent period

Before vaccination (Jan 2020)–(Dec 2020)							After vaccination (May 2021 – April 2022)							Recent period (June 2022 – Dec 2022)						
Canada	England	France	India	Italy	Netherlands	USA (WA)	Canada	England	France	India	Italy	Netherlands	USA (WA)	Canada	England	France	India	Italy	Netherlands	USA (WA)
NSP3	NSP3	NSP3	NSP3	S	NSP3	NSP3	S	S	S	S	S	S	S	S	S	S	S	S	S	S
S	S	NSP12b	S	NSP3	S	S	NSP3	NSP3	NSP3	NSP3	NSP3	NSP3	NSP3	NSP3	NSP3	NSP3	NSP3	NSP3	NSP3	NSP3
NSP12b	NSP12b	S	NSP12b	NSP12b	NSP12b	NSP12b	N	N	N	N	N	N	N	NSP4	N	N	NSP4	N	N	NSP4
5'UTR	N	5'UTR	N	N	N	5'UTR	NSP4	NSP4	NSP12b	NSP4	NSP12b	NSP12b	NSP4	N	NSP4	NSP4	NSP12b	NSP4	NSP4	N
NSP2	5'UTR	ORF3a	5'UTR	5'UTR	ORF3a	NSP13	NSP12b	NSP12b	NSP4	NSP12b	NSP4	NSP4	NSP12b	NSP5	M	M	N	M	M	M
ORF3a	NSP2	N	ORF3a	M	5'UTR	N	ORF3a	M	NSP6	ORF3a	5'UTR	ORF8	ORF3a	M	NSP5	NSP5	ORF3a	NSP5	NSP5	NSP5
N	ORF3a	NSP2	NSP14	NSP2	M	NSP2	NSP6	3'UTR	ORF3a	5'UTR	NSP6	NSP6	NSP6	ORF3a	NSP12b	NSP12b	NSP5	NSP12b	NSP12b	ORF3a
NSP13	NSP6	NSP13	NSP2	NSP16	NSP13	ORF3a	M	NSP6	ORF8	M	ORF3a	ORF3a	M	NSP12b	NSP13	ORF3a	M	ORF3a	ORF3a	NSP12b
NSP14	NSP5	M	NSP4	NSP1	NSP2	3'UTR	5'UTR	ORF3a	3'UTR	3'UTR	3'UTR	M	5'UTR	NSP15	ORF3a	3'UTR	NSP15	NSP15	NSP15	NSP15
NSP4	NSP14	NSP14	M	ORF10	NSP16	NSP14	3'UTR	5'UTR	M	ORF7a	M	3'UTR	3'UTR	ORF6	NSP15	NSP15	E	5'UTR	3'UTR	NSP6
NSP6	NSP15	NSP4	ORF8	ORF3a	ORF8	ORF8	ORF8	NSP13	5'UTR	NSP6	ORF8	5'UTR	ORF8	E	3'UTR	NSP13	NSP13	3'UTR	NSP1	ORF6
M	M	NSP6	NSP6	NSP6	NSP4	NSP4	NSP13	ORF7a	NSP5	NSP13	NSP5	NSP5	NSP13	NSP6	NSP6	NSP6	ORF6	NSP13	NSP13	NSP13
NSP15	NSP13	NSP5	3'UTR	3'UTR	NSP1	NSP15	NSP5	NSP5	NSP14	NSP5	NSP13	NSP13	NSP5	NSP13	NSP14	NSP1	NSP14	NSP6	E	3'UTR
NSP1	NSP4	NSP1	NSP13	NSP13	NSP14	NSP16	ORF7a	NSP14	NSP13	NSP14	NSP14	NSP14	NSP14	NSP14	NSP2	NSP14	NSP1	NSP14	NSP14	NSP14
3'UTR	3'UTR	3'UTR	NSP1	ORF8	NSP6	NSP5	NSP15	ORF8	NSP2	NSP15	ORF7a	NSP2	ORF7a	3'UTR	E	E	5'UTR	NSP1	NSP6	E
NSP8	NSP1	NSP15	NSP16	NSP5	ORF10	NSP6	NSP2	ORF7b	ORF7a	ORF7b	NSP2	ORF7a	NSP15	NSP1	NSP1	ORF7b	3'UTR	E	ORF7b	NSP1
NSP5	ORF8	ORF8	NSP15	NSP14	NSP15	ORF7a	NSP14	NSP15	NSP15	NSP2	NSP15	NSP15	NSP2	ORF7b	NSP8	NSP2	ORF7b	ORF7b	NSP9	ORF7b
ORF8	NSP16	NSP16	NSP8	NSP4	NSP8	M	ORF6	ORF6	ORF7b	ORF8	ORF6	ORF6	ORF6	NSP9	5'UTR	NSP9	NSP9	ORF6	ORF6	NSP9
ORF10	ORF7a	ORF7a	NSP5	NSP15	3'UTR	NSP1	NSP9	NSP2	ORF6	ORF6	ORF7b	ORF7b	ORF7b	5'UTR	ORF7b	NSP8	NSP8	NSP9	5'UTR	5'UTR
ORF7a	ORF10	ORF10	ORF7a	NSP9	NSP5	NSP8	ORF7b	NSP9	NSP1	NSP9	NSP1	NSP9	NSP1	NSP8	NSP9	5'UTR	NSP6	NSP2	NSP8	NSP2
NSP16	NSP8	NSP9	ORF6	ORF7a	NSP9	ORF6	NSP1	NSP1	NSP9	NSP1	NSP9	NSP1	NSP9	NSP2	ORF6	ORF6	NSP2	NSP8	NSP2	NSP8
NSP7	NSP7	NSP7	NSP7	NSP8	ORF7a	ORF10	E	E	E	E	E	E	E	ORF7a	ORF7a	ORF8	ORF10	ORF7a	ORF7a	ORF7a
ORF6	E	ORF7b	E	NSP7	ORF7b	NSP9	NSP16	NSP16	NSP16	NSP16	NSP16	NSP16	NSP16	ORF8	NSP16	ORF7a	ORF7a	ORF8	ORF10	NSP16
NSP9	NSP10	NSP8	ORF7b	ORF7b	E	NSP7	NSP7	NSP8	NSP8	NSP8	NSP10	NSP8	NSP8	NSP16	ORF8	ORF10	ORF8	ORF10	ORF8	ORF8
E	ORF7b	E	NSP9	NSP10	ORF6	E	NSP8	NSP10	NSP10	NSP10	NSP8	NSP10	NSP10	NSP7	NSP10	NSP7	NSP16	NSP16	NSP16	ORF10
NSP10	NSP9	ORF6	ORF10	E	NSP10	NSP10	NSP10	NSP7	ORF10	ORF10	NSP7	NSP7	ORF10	ORF10	NSP7	NSP16	NSP7	NSP7	NSP7	NSP10
ORF7b	ORF6	NSP10	NSP10	ORF6	NSP7	ORF7b	ORF10	ORF10	NSP7	NSP7	ORF10	ORF10	NSP7	NSP10	ORF10	NSP10	NSP10	NSP10	NSP10	NSP7
-	NSP12a	NSP12a	NSP12a	NSP12a	NSP12a	NSP12a	-	NSP12a	NSP12a	NSP12a	NSP12a	NSP12a	NSP12a	**-**	-	-	-	NSP12a	-	NSP12a

The findings are in line with previous studies suggesting significant purifying selection which has been the primary factor driving the SARS-CoV-2 evolution and ongoing strong positive selection observed within certain sites of the SARS-CoV-2 genome (64). In a previous study, it has been suggested that increase in the selection pressures can cause increase in the mutation rates measured (65), which is also justified in our study as both mutation rates and d*N*/d*S* ratios are increasing with time. Combined with the persistence of recurrent infections in immunocompromised individuals, this may have induced the selection of viruses with lower pathogenicity or virulence and higher transmission (66).
  - Selection pressure in SARS-CoV-2 proteins: The d*N*/d*S* ratios of the proteins in all the countries are shown in Table 5. SARS-CoV-2 proteins with the strongest positive selection pressures in the pre-vaccination period were N, ORF8, ORF3a, and ORF10. In a change from the pre-vaccination period, the post-vaccination marked substantial increase in E, ORF7a and NSP3. The recent phase sequences that E, NSP1, and NSP13 had and the highest d*N*/d*S* ratios. A continuous trend of positive selection was identified in *S* in all the three phases. Previous studies during the year 2020 have identified ORF10 to be under strong positive selection, along with ORF3a, ORF8 and N also depicting much higher number of d*N* (62, 67, 68). ORF8 is involved in host-pathogen interactions through its nine encoded proteins (69), ORF3a is involved in interference with host ion channels by encoding viroporin (70), while overexpression of ORF10 is known to downregulate IFN-1 expression, leading to suppression of the antiviral innate immune response (71). However, most viral proteins are multifunctional, and their other roles are likely to be discovered in future. ORF7a is responsible for triggering the NF-kappa B pathway and pro-inflammatory expression of cytokines (72). Also, evidence of strong positive selection pressures was observed in NSP3 from a 2021 study (73). NSP3 and its interaction with *N* is known to affect SARS-CoV-2 replication and its pathogenesis (74). In the pre-vaccination phase, NSP1 was under negative selection pressure with d*N*/d*S* < 1. Also, in a previous study where NSP1 showed negatively selected sites only and it is designed to be evolutionary conserved for its functional requirement of host ribosomal complex binding (75, 76). *E* and *S* indicated strong positive selection pressures in a 2021 study (73). *E* is involved in the expression of a small multi-functional protein that has an important role in host and virus interaction through ion-channel activity. Mutations within *E* are associated with a reduction in virulence (77, 78). NSP13 has also shown strong evidence of positive selection which may be justified due to its role in inhibiting antiviral immunity by hijacking host deubiquitinase USP13 and suppresses type I IFN response through contact with TBK1 (64, 79). *S* has shown continuous strong positive selection in our study in all the three phases as supported by other studies (64, 80). NSP1 showed a large jump in the d*N*/d*S* ratio with a drastic increase in the number of d*N* after vaccination. NSP1 is suggested to suppress the host innate immunity by interacting with the 40S ribosomal subunit (76, 81, 82). NSP9 was observed to be the most common gene having d*N*/d*S* < 1 showing purifying selection. NSP9 is said to be highly conserved and involved in viral RNA synthesis (83).

**Fig 2:**
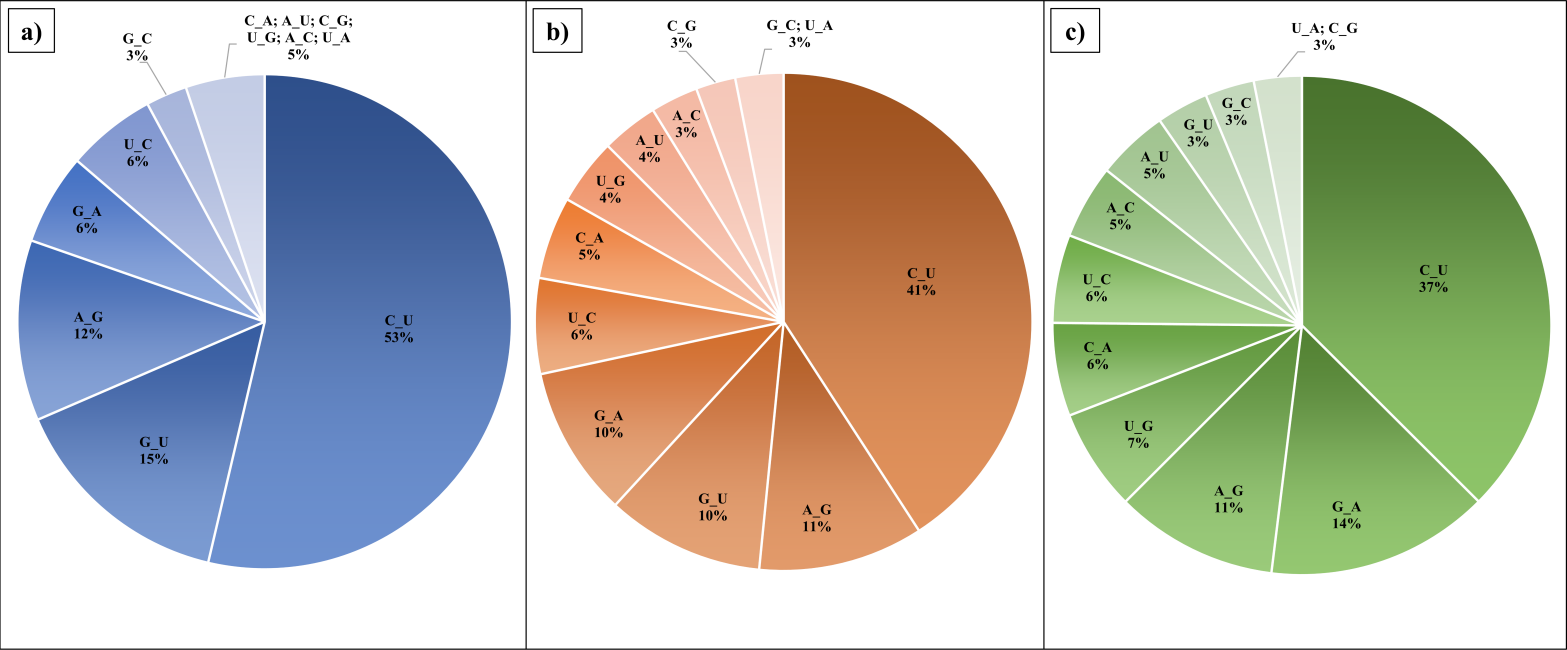
Percentage proportions of transition and transversion mutations in the countries in the three phases. (**a**) Before vaccination (pre-vaccination period);,(**b**) after vaccination (post-vaccination period), and (**c**) recent period.

- Decrease in T*i*/T*v* ratio: The estimated T*i*/T*v* ratios for each country in the pre-vaccination, post-vaccination, and the recent period are listed in Table 1. The T*i*/T*v* ratio in the pre-vaccination data set was found to be 3.77 ± 1.10. The value is in agreement with the range of T*i*/T*v* of 2.0 to 5.5 calculated in previous studies (84, 85). The T*i*/T*v* ratio decreased in the post-vaccination period to 2.00 ± 0.12 and remained similar with the recent period sequences, i.e., 2.15 ± 0.05. The differences in the mean T*i*/T*v* ratios were found to be highly statistically significant between the three phases with *P*-value of less than 0.0001 (Table 2). Figure. 1c shows the comparison of T*i*/T*v* ratios within the countries in pre-vaccination, post-vaccination, and recent period. As observed from the figure, the proportion of T*v* were found to be increased post-vaccination. As T*v* are said to have a dominant contribution toward d*N* within the genome, an increase in T*v* can be directly related to increase in d*N*. Thus, a decrease in T*i*/T*v* ratio is correlated with an increase in the d*N*/d*S* ratio. Previous studies have shown that T*i* saturate more rapidly with time in comparison with T*v*, thus causing T*i*/T*v* ratios to decline with evolutionary time (86–89).
  - Effect of T*i* and T*v* patterns on CpG ratios: The percentage of T*i* and T*v* in the pre-vaccination, post-vaccination and recent period are shown in Fig. 2a through c respectively. The most frequent T*i* and T*v* observed was C→U and G→U respectively in the pre- and post-vaccination period. The C→U and G→U substitutions result in the deterioration of CpG content. In the post-vaccination period, the C→U substitutions decreased by 12% from the pre-vaccination period, whereas in recent period, a slight decrease of 4% was observed from the post-vaccination period. In comparison with C→U, G→U substitutions deteriorated at a slower rate, i.e., 5% reduction post-vaccination and about 7% reduction in recent period from post-vaccination period. In the recent period sequences, C→U remained the most frequent T*i*, whereas U→G was the most frequent T*v*. A previous study that evaluated the sequences of the period January 2020 to March 2021 reported a drastic increase in C→U and G→U substitutions in the initial phase of SARS-CoV-2 infection, which eventually stabilized at a point of time and then decreased till March 2021 (90). Table 1 shows the %GC content in the genome sequences from each country for pre-vaccination, post vaccination and recent period. It can be observed that the %GC content slightly reduced after vaccination, whereas negligible reduction was seen in the recent period genome sequences. As a result, the overall decline in GC content is minimal and appears to be stabilizing in future. This is confirmed by another study which depicted minute variations in the CpG content in the SARS-CoV-2 genome sequences and concluded that CpG content reduced at a faster rate in the initial time of evolution which may slow down to become steady with the evolution in human host (91). Thus, these mutations were attributed to rapid evolution after transmission to the human host (92–94). The differences in the mean % GC content were found to be highly significant as tested a two-tailed *P*-value of less than 0.0001 (Table 2). It has also been reported that the CpG motifs are lost in the SARS-CoV-2 sequences, which may enable antiviral response escape through TLR7 (91, 95). Higher U→G has been reported in the SARS-CoV-2 genome sequences collected between January 2022 and July 2022 (96). This further suggested that the amount of C→U and G→U substitutions are likely to decrease upon increase in divergence indicating that some portion of these mutations are knocked out by purifying selection, irrespective of the origin of SARS-CoV-2 (90, 97). Reduction in C→U and G→U substitutions together with the increase in U→G may eventually lead to an increase in the CpG content in the SARS-CoV-2 genome. Higher CpG content has been linked to the attenuation of the virus (98, 99).

**TABLE 5:** Highest (top) to lowest (bottom) protein-wise contribution to selection pressure (d*N*/d*S* ratio) in all the countries before, after vaccination and in the recent period

Before vaccination							After vaccination							Recent period						
Canada	England	France	India	Italy	Netherlands	USA (WA)	Canada	England	France	India	Italy	Netherlands	USA (WA)	Canada	England	France	India	Italy	Netherlands	USA (WA)
ORF8	N	ORF10	ORF3a	ORF10	ORF10	ORF3a	E	E	ORF7a	ORF7a	E	E	E	E	E	E	E	E	E	E
ORF3a	ORF3a	N	N	N	ORF3a	ORF8	S	ORF7a	NSP3	E	NSP3	ORF8	ORF8	S	S	S	NSP1	NSP1	NSP1	NSP1
NSP7	NSP6	NSP7	NSP12b	S	S	N	ORF8	NSP3	S	S	ORF7a	ORF7a	S	NSP1	NSP1	NSP1	S	S	S	S
NSP8	ORF10	S	ORF8	NSP12b	NSP13	NSP13	NSP3	S	ORF8	NSP3	ORF8	S	ORF7a	N	NSP13	NSP13	N	NSP13	NSP13	NSP14
N	S	NSP13	NSP6	NSP13	N	NSP16	NSP14	ORF8	E	NSP13	S	NSP3	N	NSP13	N	N	ORF10	N	N	NSP13
ORF10	NSP12b	NSP12b	NSP8	NSP8	NSP12b	S	NSP13	NSP1	NSP1	NSP14	NSP13	NSP1	NSP13	NSP14	NSP14	ORF10	NSP8	NSP14	ORF10	N
NSP12b	NSP8	NSP4	NSP2	ORF3a	ORF7a	NSP5	ORF7a	NSP14	NSP13	NSP1	NSP1	NSP13	NSP14	NSP7	M	M	NSP4	ORF10	NSP14	ORF10
NSP16	NSP2	ORF3a	NSP15	ORF7a	NSP9	NSP12b	M	ORF10	ORF10	M	NSP4	NSP14	NSP16	ORF10	NSP6	NSP14	ORF8	M	ORF8	ORF8
S	NSP7	ORF7a	S	ORF8	NSP6	NSP2	NSP4	NSP13	NSP4	ORF8	NSP14	NSP4	M	NSP4	ORF10	NSP6	NSP13	ORF8	M	NSP4
NSP2	NSP5	ORF8	E	NSP4	NSP8	E	ORF3a	M	NSP14	NSP4	M	M	NSP1	NSP8	ORF8	NSP4	NSP14	NSP4	NSP4	M
NSP15	NSP9	E	ORF7b	NSP14	NSP10	ORF10	NSP1	NSP4	ORF3a	NSP12b	ORF3a	ORF3a	NSP4	ORF8	NSP4	ORF8	NSP6	NSP6	NSP16	NSP10
NSP6	ORF6	NSP8	NSP16	NSP5	NSP5	ORF7b	NSP6	ORF3a	M	ORF3a	NSP6	ORF10	ORF3a	M	NSP2	NSP7	M	NSP12a	NSP2	NSP7
M	ORF7a	ORF6	ORF7a	E	NSP4	NSP6	NSP16	N	N	NSP6	NSP10	NSP6	NSP10	NSP16	NSP16	NSP2	NSP16	NSP16	NSP7	NSP16
ORF7b	NSP13	NSP2	ORF10	NSP6	NSP2	ORF7a	NSP8	NSP12b	NSP6	ORF10	ORF10	N	NSP3	NSP12b	NSP12b	NSP12b	NSP2	ORF3a	NSP12b	NSP3
NSP13	E	NSP9	NSP7	NSP2	NSP7	NSP9	N	NSP16	NSP16	N	N	NSP15	NSP12b	ORF7a	NSP15	ORF3a	NSP12b	NSP12b	NSP3	NSP15
E	NSP10	NSP16	NSP5	NSP7	NSP14	NSP1	NSP15	ORF7b	NSP15	ORF7b	NSP8	NSP12b	NSP6	NSP3	ORF6	NSP15	NSP3	NSP3	NSP6	NSP8
NSP14	ORF8	NSP5	NSP13	NSP15	NSP15	NSP7	NSP12b	NSP6	NSP8	NSP15	NSP16	NSP16	ORF10	NSP2	ORF3a	NSP3	NSP15	NSP15	NSP15	ORF6
NSP5	NSP15	NSP15	NSP9	ORF6	ORF7b	NSP8	NSP10	NSP15	NSP12b	NSP2	NSP12b	ORF6	NSP8	NSP10	NSP3	NSP16	ORF6	ORF6	ORF6	NSP12b
NSP9	NSP14	NSP1	NSP4	NSP9	E	ORF6	NSP2	NSP8	ORF7b	NSP16	NSP15	ORF7b	NSP15	NSP15	NSP5	ORF6	NSP10	NSP2	ORF3a	NSP2
NSP4	NSP16	NSP10	NSP3	NSP3	NSP3	NSP4	ORF6	ORF6	ORF6	ORF6	ORF6	NSP2	ORF7b	ORF6	ORF7a	ORF7a	ORF7a	NSP7	NSP10	ORF3a
NSP10	NSP4	NSP6	NSP10	ORF7b	ORF8	NSP14	ORF10	NSP2	NSP10	NSP10	ORF7b	NSP10	ORF6	ORF3a	NSP10	NSP5	ORF3a	NSP5	NSP8	ORF7a
ORF6	ORF7b	NSP14	ORF6	NSP10	ORF6	M	ORF7b	NSP10	NSP7	NSP8	NSP2	NSP5	NSP2	NSP5	NSP7	NSP10	NSP5	NSP10	NSP5	NSP5
NSP3	M	NSP3	NSP1	NSP16	M	NSP3	NSP5	NSP5	NSP2	NSP7	NSP5	NSP8	NSP7	NSP6	NSP8	NSP8	NSP9	ORF7a	ORF7a	NSP6
NSP1	NSP3	ORF7b	NSP14	NSP1	NSP16	NSP10	NSP7	NSP7	NSP5	NSP5	NSP7	NSP7	NSP5	ORF7b	ORF7b	NSP9	ORF7b	NSP8	ORF7b	ORF7b
ORF7a	NSP1	M	M	M	NSP1	NSP15	NSP9	NSP9	NSP9	NSP9	NSP9	NSP9	NSP9	NSP9	NSP9	ORF7b	NSP7	ORF7b	NSP9	NSP9
5'UTR	5'UTR	5'UTR	5'UTR	5'UTR	5'UTR	5'UTR	5'UTR	3'UTR	3'UTR	5'UTR	5'UTR	3'UTR	5'UTR	3'UTR	3'UTR	3'UTR	3'UTR	NSP9	3'UTR	3'UTR
3'UTR	3'UTR	3'UTR	3'UTR	3'UTR	3'UTR	3'UTR	3'UTR	5'UTR	5'UTR	3'UTR	3'UTR	5'UTR	3'UTR	5'UTR	5'UTR	5'UTR	5'UTR	3'UTR	5'UTR	5'UTR
-	NSP12a	NSP12a	NSP12a	NSP12a	NSP12a	NSP12a	-	NSP12a	NSP12a	NSP12a	NSP12a	NSP12a	NSP12a	-	-	-	-	5'UTR	-	NSP12a

### Evolutionary patterns across different SARS-CoV-2 variants

The genomic mutation rates (%), d*N*/d*S* and T*i*/T*v* ratios were also determined for each SARS-CoV-2 variant (shown in Table 6).

**TABLE 6:** Data comparison between genomic and spike protein among different SARS-CoV-2 variant sequences^*a*^

Variants	No. of sequences	No. of genomic mutations	Genomic mutation rate (%)	Spike protein mutation rate (%)	Genomic d*N*/d*S* ratio	Spike d*N*/d*S* ratio	T*i*/T*v* ratio
Alpha	1,056	33,299	0.12	0.03	0.73	**14.60**	*1.68*
Beta	1,005	31,174	*0.10*	0.03	0.92	4.46	2.07
Delta	997	40,231	0.14	0.03	**1.43**	5.62	2.00
Gamma	1,003	37,597	0.13	0.04	0.60	10.75	1.81
Lambda	1,002	33,305	0.12	0.03	*0.58*	*1.42*	**2.80**
Mu	1,011	40,142	0.13	0.03	0.77	6.32	2.15
Omicron	1,135	71,411	**0.22**	**0.10**	1.01	4.46	2.00
**Overall Average**			**0.14**	**0.04**	0.86	6.8	2.07

^*a*^ The highest mutation rates, d*N*/d*S* and T*i/*T*v* ratios are shown in bold while the lowest are shown as italics.

- Mutation rate (%) analysis: The variant-wise genomic mutation rate analysis data suggest Omicron variant has the highest mutation rates among all variants (0.22%) followed by Delta (0.14%). Beta variant displayed the lowest mutation rate (0.10%). Previous studies showed Omicron to have high genomic mutation rate along with the accumulation of highest number of mutations (100–102).
  - It has been suggested that the *S* mutations are positively selected to generate new variants of SARS-CoV-2 that have improved overall fitness (66, 103). To evaluate this, the average number of mutations, mutation rates and d*N*/d*S* ratios were determined for the *S* region in each variant. The average number of *S* mutations were calculated as a percentage of total number of *S* mutations in the genome. The resulting *S* mutations percentage for Alpha, Beta, Delta, Gamma, Lambda, Mu, and Omicron were 29.4%, 30.5%, 22.7%, 33.7%, 25.3%, 26.3%, and 47.8% respectively. As seen in Table 6, the highest mutation rates in the whole genome as well as in *S* are seen in the Omicron variant. *S* mutation rates of all the variants are similar except for Omicron, which has the highest *S* mutation rate of 0.1. This has also been observed in a previous study (100). The mutation rate patterns for proteins in each SARS-CoV-2 variant is depicted in Table 7.

- d*N*/d*S* ratio analysis: As d*N*/d*S* ratios exceed unity for each SARS-CoV-2 variant, positive selection is responsible for promoting d*N* in the sequences. From Table 6, the average genomic d*N*/d*S* of all SARS-CoV-2 variants is estimated to be around 0.86 compared to the Wuhan reference strain which is similar with another study that estimated it to be 0.8446 (104). Also, it was observed that Delta variant had the highest d*N*/d*S* ratio (1.43) showing positive selection, followed by Omicron variant (1.01) showing neutral selection. Although the number of mutations is found to be higher in Omicron variant, the ratio of d*N*/d*S* is higher in the Delta variant. Delta variant was found to be highly virulent in comparison to the Omicron variant, which evolved to be more transmissible and less virulent (105–107). Therefore, the genomic d*N*/d*S* seems to correlate well with increased virulence. It has been argued that as virulence causes hindrance of transmission between hosts, d*N*/d*S* ratios may work to decrease virulence—thereby increasing transmission between hosts (108, 109).
  - Virulent genes have been observed to be highly subject to d*N* and, therefore, are under strong positive selection (110). Therefore, the protein-wise contribution to d*N*/d*S* was also determined in each of the variants and shown in Table 8. The unique genes having highest d*N*/d*S* in the highly virulent Delta variant were ORF7b and ORF7a while the unique genes contributing most to d*N*/d*S* in the Omicron variant were E, NSP14, and NSP1. The accessory protein ORF7b has been reported to mediate the cellular apoptosis caused by Tumour Necrosis Factor α (111). The ORF7a protein initiates autophagy and helps in virus replication (112). Taken together, these proteins impair the host cell immune response and cellular function (113).
  - The d*N*/d*S* ratio of *S* was greater than 1 for all the variants. This is in line with the previous findings which suggest that *S* protein coding gene had the d*N*/d*S* ratio greater than 1 in all the SARS-CoV-2 variants (114). Alpha variant *S* protein showed the highest d*N*/d*S* ratio whereas Lambda variant *S* protein had the lowest d*N*/d*S* ratio. However, d*N*/d*S* values of both genome and *S* are comparatively lower in Omicron variant. Thus, there seems to be no correlation between d*N*/d*S* ratios of *S* and genomic d*N*/d*S* ratios, virulence, or transmission. That Omicron and Delta variant have the highest and the lowest *S* mutations, respectively, was also remarked in a previous study (114). The contribution of the SARS-CoV-2 proteins toward the mutation rates and d*N*/d*S* ratios in each variant has been shown in Tables 7 and 8, respectively.
- T*i*/T*v* ratio analysis: Lambda variant was found to have the lowest d*N*/d*S* ratio but the highest T*i*/T*v* ratio. A negative correlation has previously been observed between T*i*/T*v* ratio and d*N*/d*S* ratio under positive selection in the comparative analysis of SARS-CoV, SARS-CoV-2 and MERS-CoV for all the nucleotide substitution models (24). The reason for this could be explained by the fact that T*v* substitutions favour d*N*. Previous studies have also remarked that higher number of T*i* in the Lambda variant would remark lesser d*N* (86–89).

**TABLE 7:** Highest (top) to lowest (bottom) contribution of the proteins toward the mutation rates (%) in SARS-CoV-2 variants

Alpha	Beta	Delta	Gamma	Lambda	Mu	Omicron
S	S	S	S	S	S	S
NSP3	NSP3	NSP3	NSP3	N	NSP3	NSP3
NSP12b	ORF3a	N	N	NSP3	ORF8	NSP4
ORF8	NSP2	NSP4	NSP12b	NSP4	ORF3a	N
N	5'UTR	NSP12b	3'UTR	NSP12b	3'UTR	M
NSP2	N	ORF7a	ORF3a	NSP6	NSP14	NSP5
5'UTR	NSP12b	5'UTR	NSP13	ORF8	NSP13	NSP12b
NSP6	NSP5	3'UTR	NSP6	3'UTR	N	ORF3a
3'UTR	NSP6	NSP6	NSP1	NSP5	NSP12b	ORF6
NSP13	ORF8	ORF3a	ORF8	5'UTR	NSP4	NSP15
ORF3a	E	NSP13	5'UTR	M	NSP15	NSP6
NSP4	NSP13	NSP14	NSP9	ORF3a	NSP6	3'UTR
NSP15	NSP4	M	NSP2	NSP2	5'UTR	5'UTR
NSP14	NSP14	ORF8	NSP14	NSP14	NSP10	NSP14
NSP8	M	NSP2	NSP4	NSP13	NSP2	ORF7b
NSP5	3'UTR	ORF7b	NSP15	ORF7a	NSP16	E
NSP9	NSP15	NSP9	NSP5	NSP16	ORF7a	NSP13
M	NSP16	NSP15	NSP10	NSP15	M	NSP1
ORF7a	ORF7b	NSP7	NSP16	NSP1	NSP1	NSP9
NSP16	ORF7a	NSP5	M	NSP8	NSP8	NSP10
NSP1	NSP1	NSP1	ORF7a	ORF6	NSP5	NSP2
NSP10	NSP8	NSP16	NSP8	NSP9	NSP9	NSP16
ORF10	NSP9	ORF6	E	NSP7	ORF6	ORF8
E	NSP7	NSP8	ORF7b	NSP10	NSP7	ORF7a
NSP7	NSP10	E	NSP7	ORF10	ORF7b	ORF10

**TABLE 8:** Highest (top) to lowest (bottom) contribution of the proteins toward the selection pressure (d*N*/d*S* ratios) in SARS-CoV-2 variants

Alpha	Beta	Delta	Gamma	Lambda	Mu	Omicron
ORF8	E	ORF7b	S	NSP5	ORF8	E
N	ORF3a	ORF7a	ORF8	ORF7b	ORF3a	NSP14
S	S	N	E	NSP12a	NSP6	NSP1
NSP13	N	S	ORF7b	S	S	N
ORF3a	NSP16	ORF6	ORF3a	ORF3a	NSP13	NSP6
ORF10	NSP5	M	NSP10	N	NSP4	S
NSP8	NSP12b	NSP12b	NSP13	NSP8	ORF7b	NSP13
E	ORF7a	NSP13	ORF7a	NSP3	NSP12b	NSP4
NSP10	ORF7b	ORF3a	N	NSP4	E	M
NSP4	NSP1	E	NSP6	ORF7a	NSP1	NSP16
ORF6	NSP4	NSP14	NSP4	ORF6	N	NSP8
M	NSP2	ORF10	ORF10	NSP9	ORF10	NSP3
NSP16	NSP13	ORF8	NSP8	E	NSP2	NSP15
NSP14	NSP6	NSP4	NSP15	NSP2	NSP3	ORF7a
NSP6	NSP3	NSP8	NSP16	NSP10	ORF7a	NSP12b
NSP3	ORF6	NSP3	NSP5	NSP14	NSP5	ORF3a
ORF7a	NSP15	NSP2	M	NSP6	NSP9	NSP5
NSP5	NSP14	NSP16	NSP2	NSP12b	ORF6	ORF8
NSP9	NSP9	NSP10	NSP12b	ORF10	NSP7	ORF6
ORF7b	NSP7	NSP6	ORF6	NSP1	NSP16	NSP2
NSP15	M	NSP1	NSP3	NSP15	NSP8	ORF10
NSP1	ORF8	NSP15	NSP7	NSP7	NSP15	NSP7
NSP7	NSP10	NSP7	NSP14	NSP13	M	NSP10
NSP12b	NSP8	NSP5	NSP1	NSP16	NSP14	ORF7b
NSP2	ORF10	NSP9	NSP9	ORF8	NSP10	NSP9
ORF8	E	NSP12a	NSP12a	M	NSP12a	NSP12a

### Conclusion

To the best of our knowledge, this is the only study that has compared the mutation rates, d*N*/d*S* ratios and T*i*/T*v* ratios during pre-, post-vaccination, and the recent periods in different geographical areas after the emergence of COVID-19. While individual studies find support from literature, this remains a comprehensive study on genomic parameters of SARS-CoV-2 in different regions and time periods.

The mutation rates were observed to be increased from the before vaccination period to the recent period in each country. The d*N*/d*S* ratio has increased with time, signifying accumulation of non-synonymous mutations favoring d*N*. However, the T*i*/T*v* ratio depicted significant decrease over time and may be correlated with viral attenuation as suggested earlier. Based on these parameters, the unpaired *t*-test helped in confirming that differences amongst the three phases were extremely statistically significant (*P*-value < 0.0001). As *S* is the main target for the vaccine, it has been proposed that mutations in this protein are driven by natural selection (66). While the number of mutations was observed to be increased in *S* in the Omicron variant, most of them were d*S* mutations.

A higher d*N*/d*S* ratio is also seen in another structural protein *N*. A 2020 communication proposed *N* as a vaccine target in view of its low mutation rates (115). Indeed, the current diagnostic kits also target *N* recognition. This may need to be re-evaluated in the light of the high mutation rates as well as d*N*/d*S* ratios of *N* as observed in our study. Conversely, ORF10, NSP7, and NSP10 coding genes had the lowest mutation rates across all the considered demographic regions and could further be targeted and explored for SARS-CoV-2 diagnostics and therapeutics.

Amongst the SARS-CoV-2 variants, Delta and Omicron had the highest d*N*/d*S* ratio and mutation rates respectively. The Delta variant has d*N* and correlates with high virulence in comparison with the Omicron variant, which is less virulent but has higher transmissibility. It was also observed that the pattern of highest to lowest gene-wise d*N*/d*S* ratios is unique in each variant genome.

The possible reasons for the observed increase in genomic mutation rates and selection pressure along with implications for vaccine efficacy against variants were considered. It was noted that:

- Vaccination is expected to slow down the number of infections, thus limiting the number of variants that the virus can explore (116). High rates of mutation may facilitate viral immune evasion and reduce the efficacy of the vaccine against infection and transmission, finally leading to reduced protection of the vaccine against several disease outcomes (117–119). The SARS-CoV-2 vaccines have indisputably lowered the COVID-19 disease burden in terms of infection, severity, and mortality (120–122). Therefore, it is likely that similar or higher mutation rates of the SARS-CoV-2 genome might have been recorded in the absence of a vaccine.
- The premise that increases in genomic mutation rates and selection pressures causes increased virus fitness and immune escape variants has also been debated as most of the mutations are deleterious (123, 124). It has been remarked that high mutation rates may even hinder the emergence of new highly adapted viruses as they do not allow viruses with advantageous genotypes to linger long enough to become fixed in a viral population (125, 126). Other causes for high mutation rates in RNA viruses have been explored, such as selection for more robust viral population or faster replication time (127).
- Differences in the evolutionary parameters like mutation rates and selection pressures in the SARS-CoV-2 genome before and after vaccination as observed in this study can be attributed to a range of factors associated with the virus, host immunity, or the environment. Furthermore, host immunity arising from natural causes as well as vaccination is expected to affect the evolution. The generation of new mutations in a viral population is not favored when the virus is well adapted to its surroundings since most mutations become harmful due to purifying selection. So, mutations that become more common can either be neutral and fixed by genetic drift or beneficial and fixed by positive selection (128).
- Pathogen fitness is characterized by an increased rate of spread in a population and has been described as a combination of increased infectivity, increased transmissibility, and a long infectious period. The public availability of genome sequences of SARS-CoV-2 variants provides a unique window to examine the changes in genomic parameters vis-à-vis the pathogen fitness. Pathogens can adapt to immunity by many mechanisms and such adaptation depends both on the new variants available in the environment at that time as well as their fitness in the host type. The over-representation of a variant in primed hosts indicates that it is immunity-adapted (129). modelled the evolution of the pathogen during vaccination campaigns by dividing it into two phases:
  - There is an initial phase during which most of the population is immunologically naïve. The first short phase favors the selection of generalist immunity-adapted variants that are equally effective against naive and primed individuals.
  - During the later second phase, most of the population acquires immunity either by natural infection or vaccination. This second phase favors specialist variants immunity-adapted to primed hosts.

The alpha, delta, and probably omicron can be classified as generalists based on epidemiological studies. It is predicted they would have spread regardless of vaccination. In future, specialist variants are predicted to appear, whereas immune-facilitated variants are rare (129–131). However, the duration required by the virus to acquire the number of mutations required for adaptation to the natural or vaccine acquired immunity cannot be predicted *a priori*.

- Vaccine-driven evolution is noted to occur in pathogens where its effects do not suppress infection, replication, or transmission sufficiently (132). Recent evidence for SARS-CoV-2 vaccines shows that the vaccine is not 100% effective against infection and disease severity. Furthermore, this effect has waned with time and emergence of new variants like omicron (130). While an array of diverse sub-lineages of the omicron variant have now emerged with the BQ and XBB sub-variants showing increased evasion of neutralizing antibodies (133), COVID-19 vaccines also elicit a specific T-cell response that may have an additional role in host protection (134). Parallels have also been made between these and the human influenza vaccines that are seasonally updated due to ongoing antigenic drift (129, 134). Most of the novel variants, in this case, remain partially inhibited by vaccination (135).
- While the currently approved SARS-CoV-2 vaccines continue to provide significant protection against severe disease and death (136), continuous monitoring and tracking for emergence of new fitter SARS-CoV-2 variants combined with molecular epidemiological surveillance is required.

## Data Availability

Raw data were generated from GISAID. Derived data supporting the findings of this study are available from the corresponding author S.B. on request. The findings of this study for pre-vaccination period are based on metadata associated with 34,970 sequences available on GISAID up to 31 December 2020 and accessible at doi:10.55876/gis8.230517nt. The findings of this study for post-vaccination period are based on metadata associated with 36,020 sequences available on GISAID from 15 May 2021 to 15 June 2022 and accessible at doi:10.55876/gis8.230517va. The findings of this study for recent period are based on metadata associated with 3,880 sequences available on GISAID from 15 June 2022 to 05 December 2022 and accessible at doi:10.55876/gis8.230518oq. The findings of this study for pre-vaccination period are based on metadata associated with 7,209 sequences available on GISAID from 30 December 2019 to 09 January 2022 and accessible at doi:10.55876/gis8.230518xv.
